# Comprehensive multi-omics analysis reveals a combination of lncRNAs that synergistically regulate glycolysis and immunotherapeutic effects in renal clear cell carcinoma

**DOI:** 10.18632/aging.206069

**Published:** 2024-08-19

**Authors:** Yuchen Li, Bowen Hou, Yan Xu, Hongze Li, Yuyan Zhu, Chuize Kong

**Affiliations:** 1 Department of Urology, The First Hospital of China Medical University, Shenyang 110001, Liaoning, China

**Keywords:** clear cell renal cell carcinoma, glycolysis, prognosis, immunotherapy efficacy, molecular docking

## Abstract

Background: Clear cell renal carcinoma is a common urological malignancy with poor prognosis and treatment outcomes. lncRNAs are important in metabolic reprogramming and the tumor immune microenvironment, but their role in clear cell renal carcinoma is unclear.

Methods: Renal clear cell carcinoma sample data from The Cancer Genome Atlas was used to establish a new risk profile by glycolysis-associated lncRNAs via machine learning. Risk profile-associated column-line plots were constructed to provide a quantitative tool for clinical practice. Patients with renal clear cell carcinoma were divided into high- and low-risk groups. Clinical features, tumor immune microenvironments, and immunotherapy responses were systematically analyzed. We experimentally confirmed the role of LINC01138 and LINC01605 in renal clear cell carcinoma.

Results: The risk profile, consisting of LUCAT1, LINC01138, LINC01605, and HOTAIR, reliably predicted survival in patients with renal clear cell carcinoma and was validated in multiple external datasets. The high-risk group presented higher levels of immune cell infiltration and better immunotherapy responses than the low-risk group. LINC01138 and LINC01605 knockdown inhibited the proliferation of renal clear cell carcinoma.

Conclusions: The identified risk profiles can accurately assess the prognosis of patients with clear cell renal carcinoma and identify patient populations that would benefit from immunotherapy, providing valuable insights and therapeutic targets for the clinical management of clear cell renal carcinoma.

## INTRODUCTION

Glycolysis is a prevalent characteristic of the tumor microenvironment and is crucial to tumor progression [1]. The primary mechanism by which oncogenic signaling increases the activity of glycolytic enzymes is through post-translational modifications or expression enhancements. Aerobic glycolysis converts glucose to lactate, a byproduct that facilitates multiple mechanisms of tumor growth and metastasis [2]. Additionally, glycolysis affects the tumor immune microenvironment, wherein tumor cells proliferate in a hypoxic setting while immune cells are inhibited, thereby facilitating the tumor cells’ evasion of the immune system [3]. As a result, glycolysis and the immune microenvironment are intricately intertwined. Long-stranded noncoding RNAs (lncRNAs) are transcripts exceeding 200 nucleotides in length and failing to encode proteins [4], which can influence the tumor immune microenvironment and modulate the epigenetic, transcriptional, and post-transcriptional mechanisms of genes in numerous pathological processes [5, 6]. Proliferation, metastasis, and an unfavorable prognosis all correlate with lncRNA expression in clear cell renal carcinoma (ccRCC) [7, 8]. By deciphering the molecular mechanisms of lncRNA in the development and progression of ccRCC, new therapeutic targets for ccRCC can be identified. An increasing number of studies have demonstrated the regulatory functions of lncRNAs in the immune microenvironment and metabolic reprogramming of tumor cells. However, no reports have been found of lncRNA associated combinations affecting ccRCC. Using machine learning and multi-omics, we identified glycolysis-associated lncRNAs (GRLs) and developed a prognostic signature to evaluate their utility in predicting treatment response and prognosis in patients with ccRCC. Additionally, we established that two HRLs (LINC01138 and LINC01605) substantially affected the proliferation of ccRCC cells. In summary, our research indicates that GRLs have the capability to forecast prognostic risk, chemotherapeutic and immunotherapeutic efficacy, and their involvement in tumor immune infiltration among patients with ccRCC.

## MATERIALS AND METHODS

### Data sources

From the TCGA database (https://portal.gdc.cancer.gov/) [9], we extracted transcriptomic data, clinical data, and somatic mutation data of ccRCC patients; patients with inadequate information or unknown survival status were excluded. A collection of lncRNAs exhibiting differential expression in hypoxia and glycolysis experiments was obtained from PubMed. By intersecting these lncRNAs with those expressed in TCGA patients, 357 GRLs were obtained (Supplementary Figure 1).

### Bioinformatics analysis

Differential analysis of ccRCC and surrounding normal tissues was performed using the R package “limma” with a cutoff of log2 fold change (logFC) >1 and an adjusted false discovery rate (FDR) <0.05 [10]. Heatmaps were visualized using the R package “pheatmap” [11]. The R packages “rms” and “regplot” were used to plot column line plots and calibration curves.

### Risk model construction and validation

A 3:7 ratio was employed to arbitrarily divide all TCGA samples between a validation dataset (n=156) and a training dataset (n=374). In order to forecast the prognosis of ccRCC patients, a prognostic model based on GRLs was constructed utilizing the training dataset. The model was validated using the validation dataset in accordance with the risk score formula utilized in the training dataset. Then, four GRLs with the most accurate prognostic values were obtained, and multivariate Cox analysis, last absolute shrinkage and selection operator (LASSO), and univariate Cox regression were used to construct the GRL model. For each sample, the risk score formula was as follows: Coefficient (lncRNA1) × Expr(lncRNA1) + Coefficient (lncRNA2) × Expr(lncRNA2) +...... + Coefficient (lncRNAn) × Expr(lncRNAn) = risk score. In the context where Expr(lncRNA) denotes the expression of lncRNA and Coef(lncRNA) signifies the regression coefficient of lncRNA.

### Immunomicroenvironment analysis

For each sample, the stroma score, ESTIMATE score, and immune cell score were computed utilizing the R package “ESTIMATE” [12]. Using the CIBERSORT algorithm (https://cibersort.stanford.edu/), the proportion of 22 forms of immune infiltrating cells was computed [13]. The algorithm known as Single Sample Gene Set Enrichment Analysis (ssGSEA) was implemented in order to determine the proportion of infiltrating immune cells. Additionally, immunoglobulin correlation analysis was conducted utilizing the software applications CIBERSORT-ABS, QUANTISEQ, MCPCOUNTER, and EPIC.

### Gene mutation analysis

We calculated the tumor mutation burden (TMB) of each patient and compared it between high-risk and low-risk groups, and then plotted a waterfall plot using the R package “Maftools”. We also analyzed the top 20 mutated genes in the high-risk and low-risk groups for mutual exclusion and synergy.

### Chemotherapy response and immunotherapy response

We downloaded gene expression data of cancer cells to various drugs from the Tumor Pharmacogenetic Multi-Omics (GDSC) database (https://www.cancerrxgene.org/) [14] and calculated IC_50_ values to assess the patients’ response to chemotherapeutic drugs.

### Construction of a competitive endogenous RNA network

The miRNAs associated with ccRCC were initially identified using the HMDD online tool [15] accessible at http://www.cuilab.cn/hmdd/. Then, HRL was combined with potential target miRNAs for prediction with the restriction of miRNA using the DIANA-LncBase online web tool (https://diana.e-ce.uth.gr/lncbasev3/home) [16]. Species: Homo sapiens; Conf. Level: High. miRTarBase [17], an online web tool accessible at https://mirtarbase.cuhk.edu.cn/, was subsequently utilized to forecast the miRNAs. This prediction was restricted to potential target mRNAs of miRNAs with a minimum of three validation techniques. Additionally, the RNA-binding protein (RBP) of HRL was predicted by employing the ENCORI online instrument (https://starbase.sysu.edu.cn/) [18]. In conclusion, ceRNA networks were developed utilizing the Cytoscape software.

### Cell counting Kit-8 (CCK8) cell activity assay

Take cells in good growth condition to prepare a certain concentration of cell suspension, 100ul per well was added into 96-well cell culture plate. Take 10ul CCK-8 solution and add it to 96-well cell culture plate, continue incubation in 37° C incubator for 0.5-4 hours. Absorbance was detected at a single wavelength of 450 nm.

### Statistical analysis

Survival curves were plotted using the Kaplan-Meier method to compare the differences in survival between the two groups, and receiver operating characteristic (ROC) curves, one-way and multifactorial Cox analyses were used to evaluate the prognostic value of the characteristics. Spearman correlation analysis was used to assess correlation. p-value ≤ 0.05 was considered statistically significant. All statistical analyses were performed with R.

### Data availability statement

All data utilized in this study are included in this article and all data supporting the findings of this study are available on reasonable request from the corresponding author.

## RESULTS

### Association of risk assessment models with prognosis and clinical features of ccRCC

**Figure 1:**
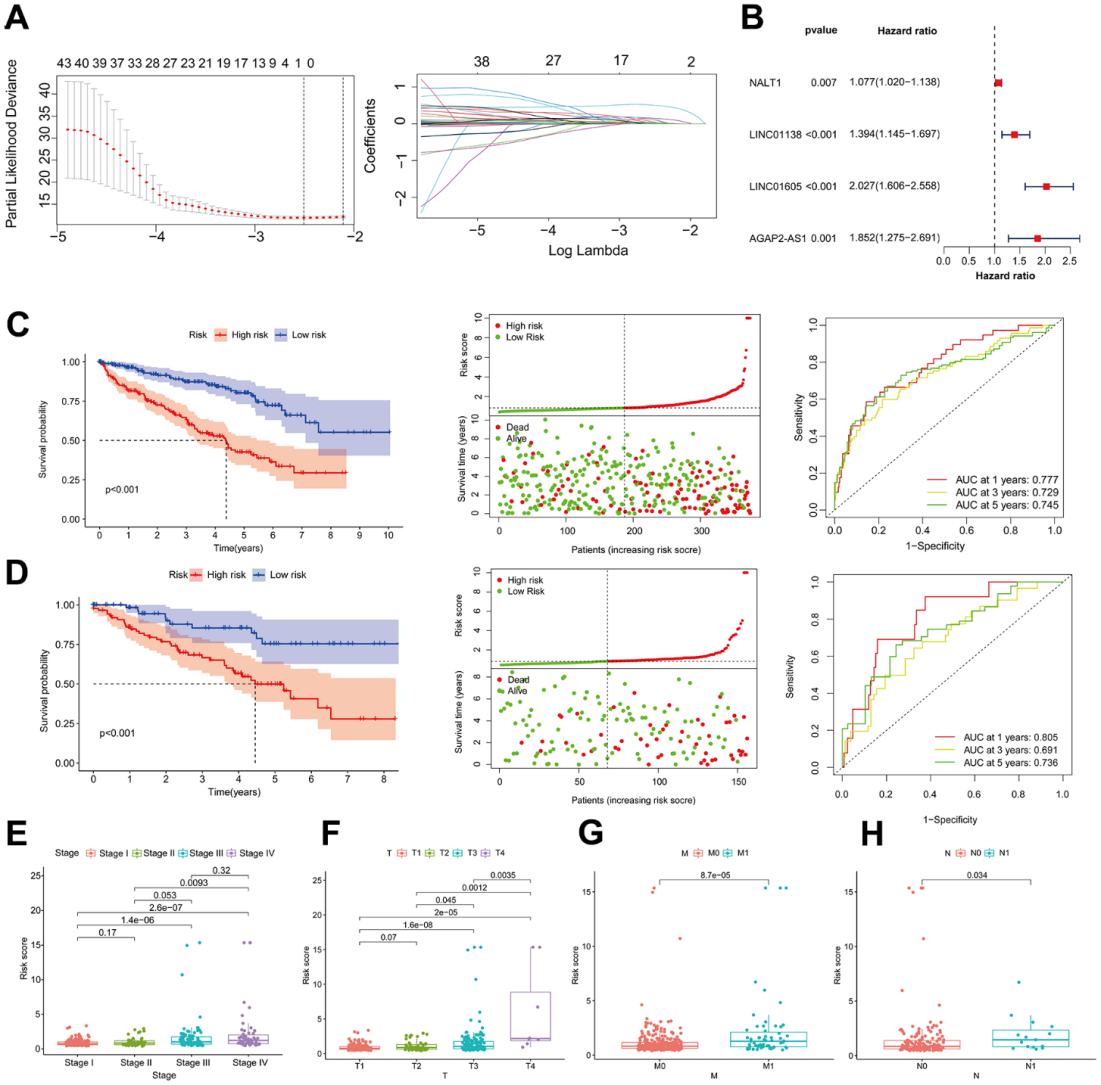
**Association of risk assessment models with prognosis and clinical features of ccRCC.** (**A**) Lasso regression analysis identifying the most robust HRLs. (**B**) Forest plot of the four GRLs in the multifactor Cox regression model. Kaplan-Meier curves, survival status and ROC curves between high and low risk groups in the training dataset (**C**) and validation dataset (**D**). Differences in risk scores between different stage (**E**), T (**F**), M (**G**) and N (**H**) strata in the training dataset.

ccRCC-expressed mRNAs were initially isolated from the TCGA database using lncRNAs; in total, 4873 lncRNAs were screened. We subsequently compiled differentially expressed lncRNAs [19, 20] in PubMed for glycolysis experiments. By measuring the intersection of the two, 357 GRLs were obtained. The risk model incorporated four GRLs (LUCAT1, LINC01138, LINC01605, and HOTAIR) that were derived via univariate Cox analysis, Lasso analysis, and multivariate Cox analysis, respectively (Figure 1A, 1B).

We randomized ccRCC patients in a 3:7 ratio between a validation dataset (n=156) and a training dataset (n=374), after excluding patients without survival data. Using the model equation, patients with ccRCC were classified into high-risk and low-risk groups. The training dataset revealed a notable disparity in the overall survival rate between patients in the low-risk and high-risk groups. Specifically, as the risk score increased, there was a corresponding rise in the number of patient fatalities. As shown in Figure 1C, the area under the curve (AUC) of the risk score utilized by the ROC curve to predict the survival of ccRCC patients one, three, and five years from now was 0.777, 0.729, and 0.745, respectively. The results presented earlier were verified using the validation dataset (Figure 1D). The examination of clinical biomarkers associated with ccRCC and their correlation with risk scores revealed statistically significant variations in risk scores across the patient strata labeled STAGE (Figure 1E), T (Figure 1F), M (Figure 1G), and N (Figure 1H). The correlation between risk profiles generated by GRLs and clinical characteristics of ccRCC suggests that they can accurately predict the prognosis of ccRCC patients.

### Risk score as an independent risk factor for ccRCC patients

**Figure 2:**
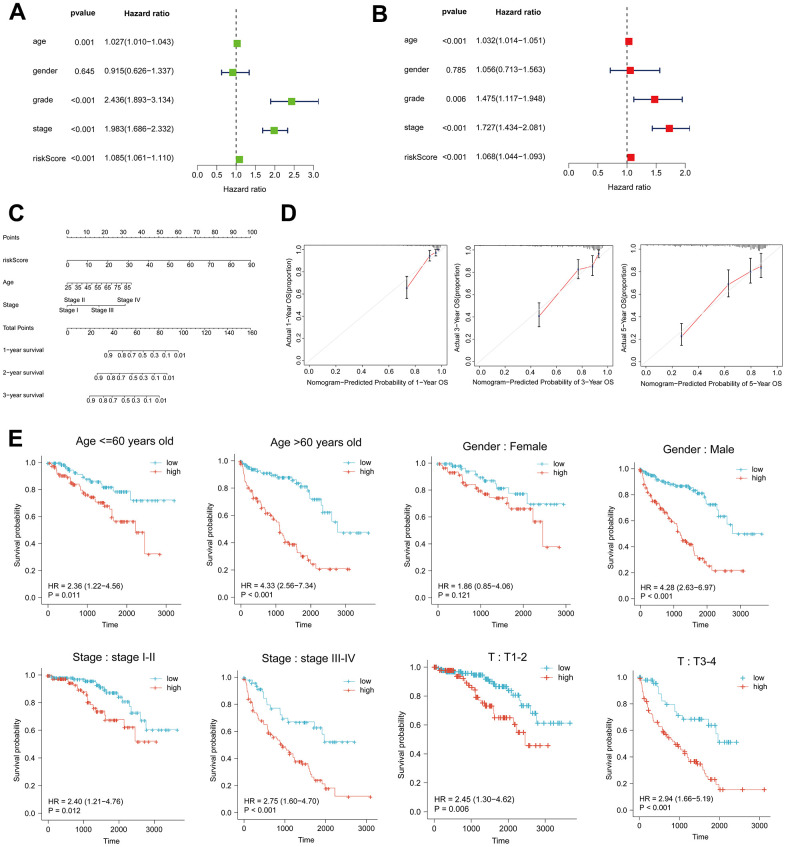
**Risk score as an independent risk factor for ccRCC patients.** (**A**, **B**) Forest plots of univariate and multivariate Cox regression analysis. (**C**) Column line plot created based on age, tumor stage and risk score. (**D**) Calibration curves at 1, 3, and 5 years for the column-line plots. (**E**) Kaplan-Meier curves for high- and low-risk patients between subgroups of ccRCC patients by age, sex, clinical stage, and T.

Univariate and multivariate Cox regression analyses were employed to examine the correlation between clinical characteristics and overall survival. According to the findings of the multivariate Cox analysis, age, tumor grade, stage, and risk score were identified as distinct prognostic factors in patients with ccRCC (Figure 2A, 2B). A column-line graph was generated using the obtained results, with the majority of the total score’s values represented by the risk score (Figure 2C). The column-line diagrams for the 1-, 3-, and 5-year calibration curves demonstrated that the predicted values corresponded precisely to the observed survival probability (Figure 2D). We further subdivided the clinical characteristics into distinct subgroups in order to determine whether HRL characteristics among distinct subgroups of ccRCC patients had prognostic value. The findings of the analysis indicated that patients aged 60 years or older and those in the HRL group had a markedly unfavorable prognosis (p<0.05). Within the gender subgroups, an analysis of the survival curves for males revealed a significant disparity between the high-risk and low-risk groups (p<0.05), whereas no statistically significant distinction was observed in overall survival between the two groups (p>0.05) for females. In the subgroups T1-2 rather than T3-4 and stage I-II compared to stage III-IV, patients with higher risk scores had a worse prognosis (p0.05) (Figure 2E). Furthermore, the survival curves between tumor grades, specifically the N and M subgroups, were examined (Supplementary Figure 2).

### High risk score suggests high immune infiltration

**Figure 3:**
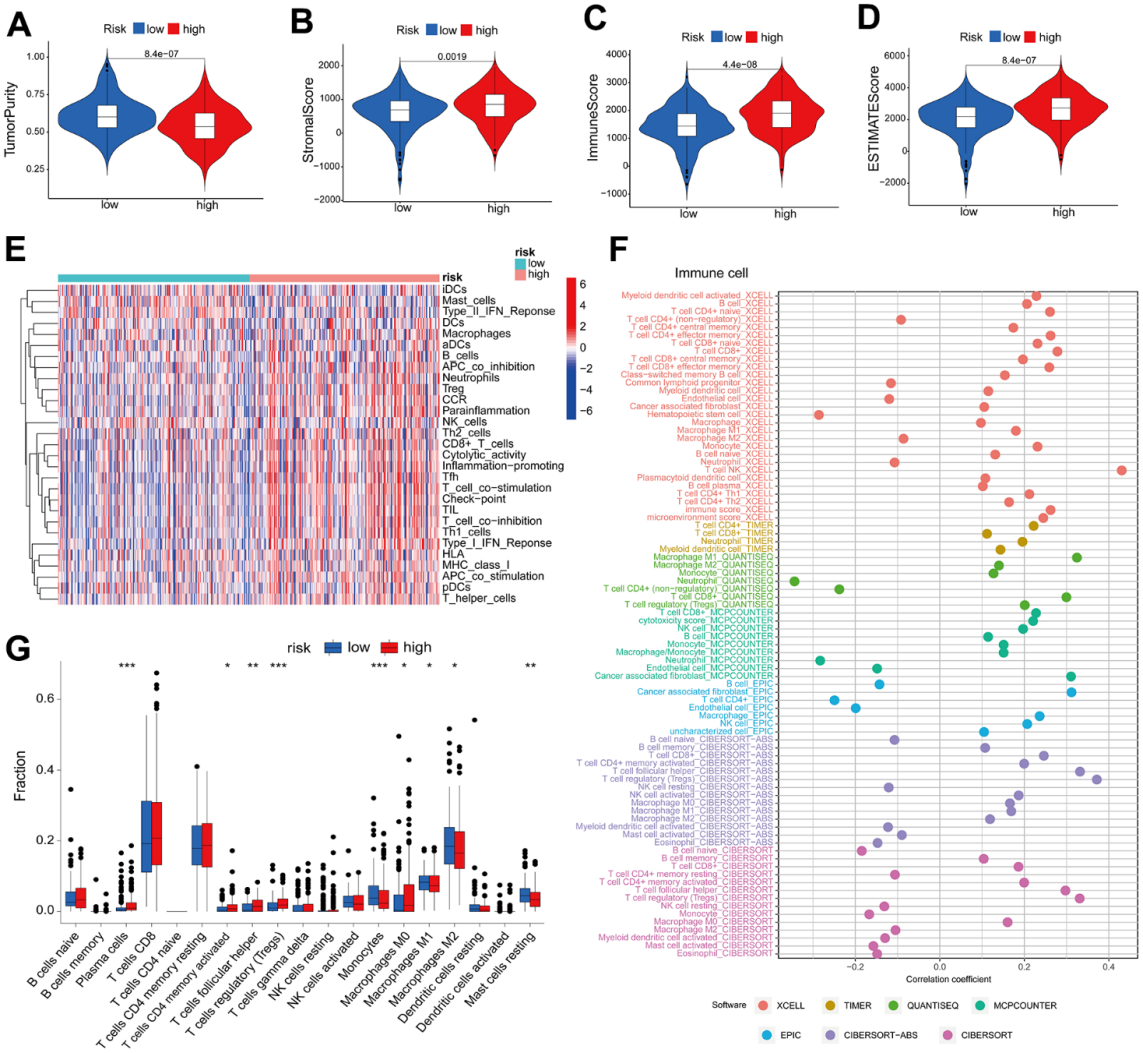
**High risk score suggests high immune infiltration.** Relationship between risk scores and tumor purity (**A**), stroma score (**B**), immune score (**C**), and ESTIMATE score (**D**). (**E**) Heatmap of the abundance of immune cells in the high-risk and low-risk groups. (**F**) Risk score and immune cell correlation. Different colors represent different algorithms. (**G**) Proportion of immune cells in the high-risk and low-risk groups. *p < 0.05, **p < 0.01, ***p < 0.001.

The tumor immune microenvironment is correlated with the prognosis of patients diagnosed with ccRCC [21]. The present study investigated the correlation between the ccRCC risk score and the immune microenvironment as measured by the ESTIMATE score. The findings revealed that tumors with a high risk score exhibited reduced purity (Figure 3A). Furthermore, there was a significant positive correlation between the risk score and the stromal score (Figure 3B), immune score (Figure 3C), and ESTIMATE score (Figure 3D) (p<0.05). The ssGSEA algorithm was utilized to estimate immune cell infiltration; the heatmap (Figure 3E) revealed that the high-risk group exhibited a significantly greater degree of immune infiltration in comparison to the low-risk group. In addition, we computed the correlation between risk scores and immune cells using seven software algorithms; the results indicated that the majority of immune cells exhibited a positive correlation with risk scores (Figure 3F). Furthermore, immunosuppressive cells (including regulatory T cells, follicular helper T cells, and M0-type macrophages) were found to be extensively infiltrated in the high-risk group, as indicated by the CIBERSORT algorithm (Figure 3G). High risk scores are indicative of low tumor purity and high immune infiltration, according to these findings, which suggest a correlation between risk profiles and immune infiltration.

### Immunotherapy

**Figure 4:**
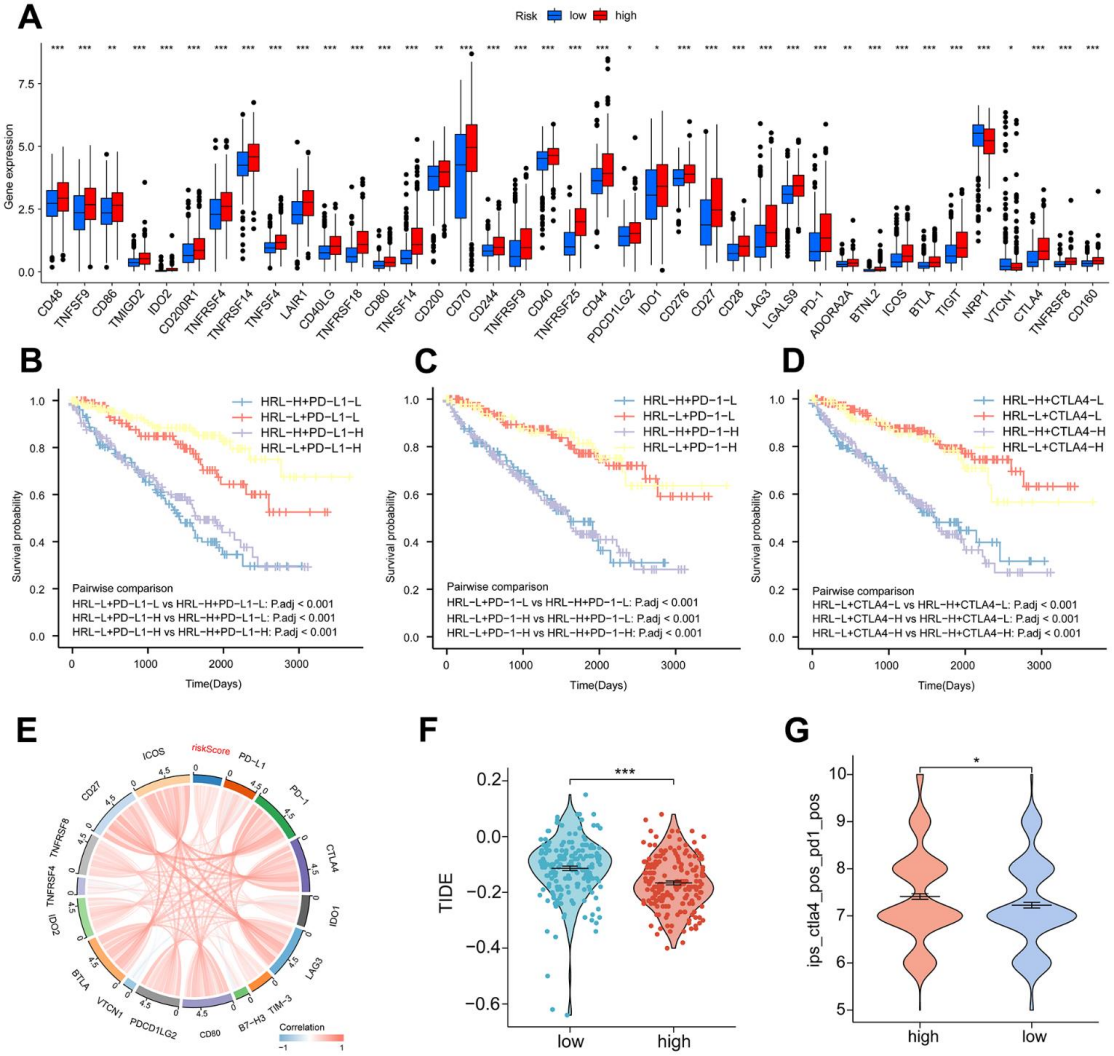
**Immunotherapy.** (**A**) Proportion of common immune checkpoints between high- and low-risk groups. patient survival curves between HRL characteristics and PD-L1 (**B**), PD-1 (**C**), and CTLA4 (**D**) stratification. (**E**) Correlation of immune checkpoint genes with risk scores. (**F**) TIDE scores in the high-risk and low-risk groups. (**G**) IPS scores for PD-1 and CTLA4 co-blockers in the high-risk and low-risk groups. *p < 0.05, **p < 0.01, ***p < 0.001.

Following the exploration of the correlation between immune checkpoint inhibitors (ICIs) and risk stratification, immunotherapy emerged as a promising therapeutic approach in the treatment of cancer. A considerable proportion of immune checkpoint expression exhibited a statistically significant increase in the high-risk group relative to the low-risk group (p<0.05) (Figure 4A). Patients with low PD-L1 expression and a low risk score had a significantly improved prognosis than those with a high risk score and low PD-L1 expression (Figure 4B). Survival was longer for patients in the low-risk group with high PD-L1 expression compared to those in the high-risk group with high PD-L1 expression (Figure 4B). In a similar fashion, the expression levels of the immune checkpoints PD-1 and CTLA4 varied significantly, with the high-risk group exhibiting a diminished overall survival rate (Figure 4C, 4D). The findings of our correlation analysis between the main immune checkpoints and the risk score indicated that the risk score was negatively correlated with VTCN1 and TNFRSF4, while the majority of the immune checks exhibited a positive correlation (Figure 4E). The application of the TIDE scoring algorithm revealed that the high-risk group exhibited a notably elevated TIDE score, indicating that immunotherapy yielded suboptimal outcomes for patients in this group as compared to those in the low-risk group (Figure 4F). Co-blockers of PD1 and CTLA4 achieved higher scores in the high-risk group (Figure 4G). According to these findings, risk scores are significantly correlated with immunotherapy in patients with ccRCC.

### LINC01138 and LINC01605 are associated with clinical staging and immune infiltration

**Figure 5:**
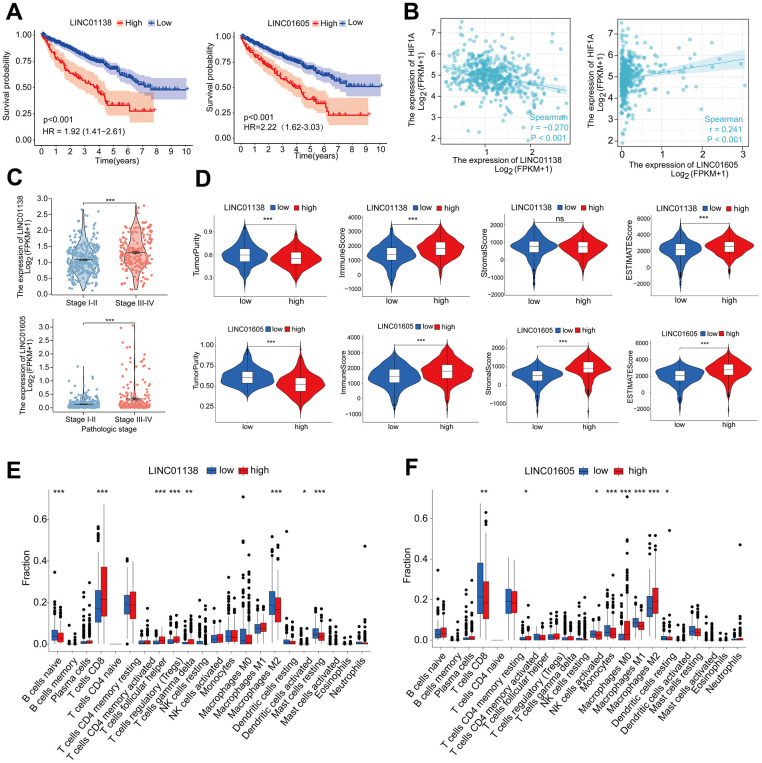
**LINC01138 and LINC01605 are associated with clinical staging and immune infiltration.** (**A**) Kaplan-Meier curves of LINC01138 and LINC01605 expression in ccRCC patients. (**B**) Correlation between LINC01138 and LINC01605 expression levels and HIF1A expression levels in ccRCC sample tissues. (**C**) Relationship between LINC01138 and LINC01605 expression and clinical stage. (**D**) Proportion of tumor purity, immune score, stroma score and ESTIMATE score at different LINC01138 and LINC01605 expression levels. (**E**) Proportion of immune cells at different LINC01138 expression levels. (**F**) Proportion of immune cells at different LINC01605 expression levels. *p < 0.05, **p < 0.01, ***p < 0.001.

We selected two risky HRLs (LINC01138 and LINC01605) in our model to further investigate the role of HRLs in ccRCC. patients with high expression of LINC01138 and LINC01605 had a poorer prognosis (Figure 5A). Correlation analysis of the expression levels of HIF1A and LINC01138 and LINC01605 in ccRCC tissue samples revealed that the two GRLs were significantly correlated with HIF1A, LINC01138 was negatively correlated with HIF1A, and LINC01605 was positively correlated with HIF1A (Figure 5B). In addition, we also found that LINC01138 and LINC01605 were significantly correlated with ccRCC clinical stage, and both HRLs had significantly elevated expression at stage III- IV, both of which predicted the prognosis and clinical progression of patients (Figure 5C).

In the tumor immune microenvironment, high expression of LINC01138 and LINC01605 showed low tumor purity, high immune score, high stromal score and high ESTIMATE score (Figure 5D). We assessed the ratio of immune cells in the high and low expression groups of the two HRLs by the CIBERSORT algorithm, in which the LINC01138 high expression group had elevated levels of CD8 T-cells and reduced levels of M2-type macrophages (Figure 5E). In contrast, the LINC01605 high expression group had significantly lower levels of CD8 T cells and significantly higher levels of M2 type macrophages (Figure 5F).

### Analysis of the molecular mechanisms of LINC01138 and LINC01605

**Figure 6:**
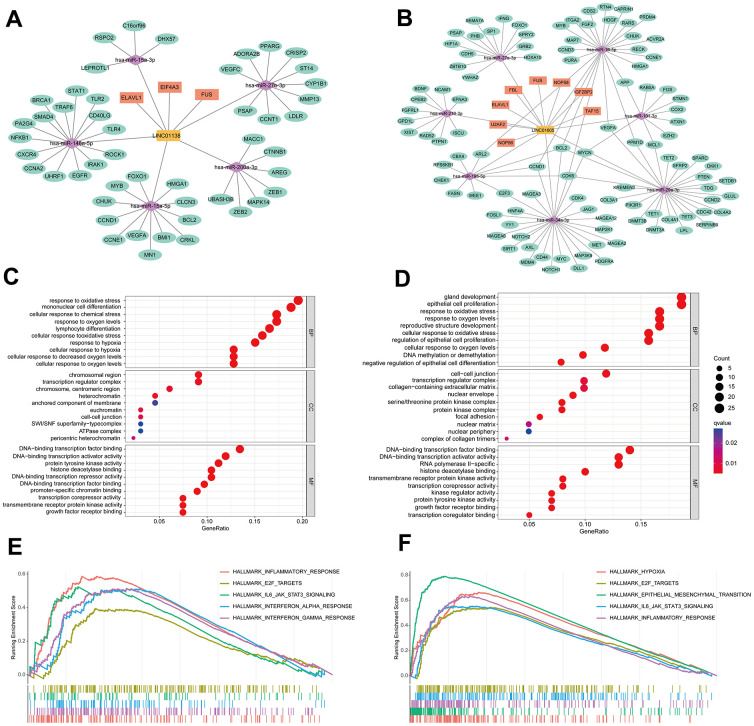
**Analysis of the molecular mechanisms of LINC01138 and LINC01605.** The ceRNA network maps of LINC01138 (**A**) and LINC01605 (**B**). mRNA enrichment analysis of LINC01138 (**C**) and LINC01605 (**D**) predicted mRNAs. GSEA analysis of differential genes between high LINC01138 (**E**) and LINC01605 (**F**) expression groups.

We identified the ceRNA networks of LINC01138 and LINC01605 using bioinformatics in order to investigate further the role of GRLs in the pathogenesis of ccRCC. We compiled ccRCC miRNAs and RBPs and hypothesized that LINC01138 and LINC01605 play interrelated functions. Following the anticipation of potential target mRNAs for miRNAs, the ceRNA networks associated with LINC01138 and LINC01605 were assembled (Figure 6A, 6B). GO enrichment analysis revealed that mRNAs that targeted LINC01138 were associated with transcriptional activation, glycolytic response, oxidative stress, and DNA transcription factor binding (Figure 6C). As shown in Figure 6D, the mRNA targeting LINC01605 was linked to oxidative stress, DNA transcriptional activation, and proliferation of epithelial cells. Following this, GSEA analysis was conducted on the groups exhibiting high LINC01138 and LINC01605 expression. The results revealed that the high LINC01138 expression group exhibited enrichment in the interferon-α signaling pathway, E2F, IL6-JAK-STAT3, and inflammatory response (Figure 6E). In the group with high LINC01605 expression, the glycolytic pathway, E2F, EMT, IL6-JAK-STAT3 signaling pathway, and inflammatory response were enriched (Figure 6F). LINC01138 and LINC01605 may be involved in the regulation of the E2F and IL6-JAK-STAT3 signaling pathways in a glycolytic environment, according to these findings.

### Knockdown of LINC01138 and LINC01605 inhibits renal clear cell carcinoma cell proliferation

**Figure 7:**
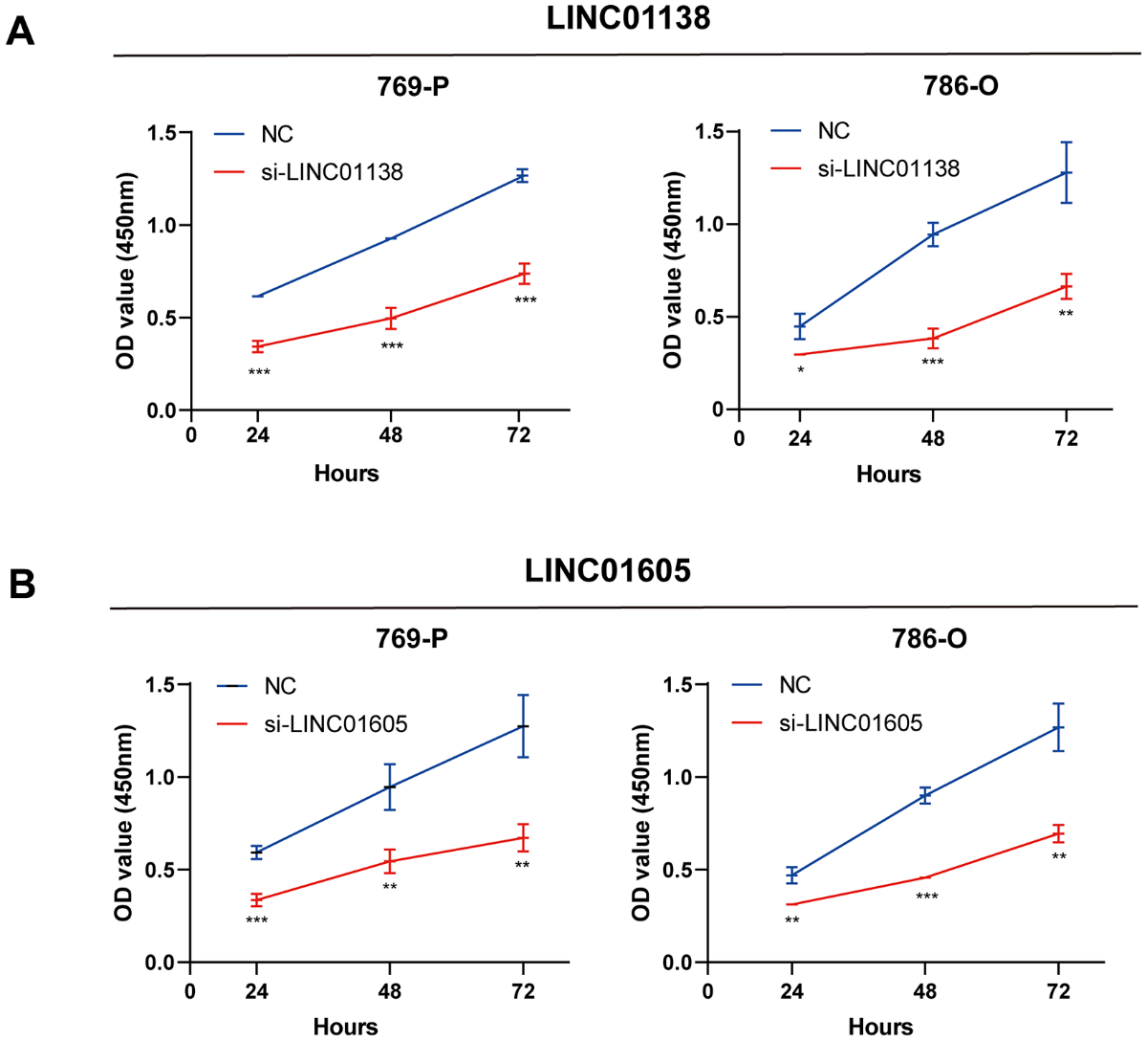
**Knockdown of LINC01138 and LINC01605 inhibits renal clear cell carcinoma cell proliferation.** Knockdown of LINC01138 and LINC01605 inhibited the proliferation ability of renal clear cell carcinoma cells. (**A**, **B**) CCK8 viability assay of 769-P and 786-O cells after transfection of si-LINC01138 and si-LINC01605. Note * p < 0.05, **p < 0.01, ***p < 0.001.

We designed siRNAs for LINC01138 and LINC01605 to silence the expression of LINC01138 and LINC01605 in human renal clear cell carcinoma cell lines 769-P and 786-O cells to investigate the roles of LINC01138 and LINC01605 in renal clear cell carcinoma. CCK8 experiments were performed by transfecting 769-P and 786-O cells with si-LINC01138 and si-LINC01605, respectively. The results of CCK8 experiments showed that the proliferative capacity of 769-P and 786-O cells in the si-LINC01138 and si-LINC01605 groups was significantly lower than that of the NC group at 24, 48, and 72 h (Figure 7A, 7B).

## DISCUSSION

Glycolysis is an important anticancer defense mechanism and therapeutic target, and the combined action of its inducers and immune checkpoint inhibitors substantially improves the therapeutic outcome for tumor patients, according to a growing body of research. Based on data from a public database, we constructed a risk signature for four GRLs (LUCAT1, LINC01138, LINC01605, and HOTAIR). In patients with ccRCC, prognosis and immunotherapy outcomes can be reliably predicted through the utilization of risk profiles. To ascertain the correlation between risk profiles and ccRCC, we initially employed gene set enrichment analysis to identify a strong association between the high-risk group and immune cells (e.g., CD8+ T cells, NK cells, and B cells). Then, using the CIBERSORT, ESTIMATE, and ssGSEA algorithms, we determined that patients in the high-risk group did, in fact, exhibit elevated levels of immune infiltration. By comparing the expression levels of the majority of common immune checkpoints, MHA molecules, cytokines, and receptors in the high-risk profile group to those in the low-risk profile group, we also discovered that these factors were significantly upregulated in the high-risk profile group. Furthermore, TMB and MSI are significant predictive factors in patient immunotherapy. Additionally, elevated levels of TMB and MSI were identified in the high-risk profile cohort. High immune cell infiltration and immune checkpoint expression may be “hot” tumor characteristics that are amenable to immunotherapy, according to the findings of the present study. The risk profile is formulated by incorporating four genes, HOTAIR, LUCAT1, LINC01138, and LINC01605. These genes have progressively demonstrated regulatory functions in glycolysis. For instance, LUCAT1 facilitates glycolysis and metastasis of lung adenocarcinoma cells by functioning as a competing endogenous RNA that regulates the miR-4316/VEGFA axis [22]. The lncRNA LINC01138 functions as an oncogenic driver; its silencing inhibits aerobic glycolysis via regulation of the microRNA-375/SP1 axis, thereby decreasing glioma cell proliferation [23]. In triple negative breast cancer cell lines, LINC01605 knockdown prevented tumor formation and migration *in vivo* by inhibiting aerobic glycolysis via lactate dehydrogenase A [24]. Knockdown of HOTAIR in hypoxia-treated hepatocellular carcinoma cells inhibits glycolysis via regulation of miR-130a-3p and HIF1A [25], a novel glycolysis mechanism in hepatocellular carcinoma. Through *in vitro* experiments, we confirmed that LINC01138 and LINC01605 depletion significantly inhibited ccRCC proliferation. In addition, small-molecule inhibitors that target lncRNAs associated with glycolysis represent a potential therapeutic approach for modulating the glycolytic process and enhancing the immunotherapeutic effects on tumor cells. While the risk profile we have developed exhibits high accuracy in predicting chemosensitivity and immunotherapeutic efficacy, in addition to ccRCC prognosis, it can also be utilized to forecast the prognosis and immunotherapeutic efficacy of numerous other types of cancer. However, this study has some limitations. First, we acquired the data used in our analysis from publicly accessible databases, which could potentially introduce bias into the process of case selection. Future *in vivo* and *in vitro* investigations are required to validate the precise molecular mechanisms by which genes that construct risk profiles for the progression of renal clear cell carcinoma operate.

## CONCLUSIONS

In this study, we identified four glycolysis-associated lncRNAs (LUCAT1, LINC01138, LINC01605, and HOTAIR) by comprehensive multi-omics analysis and *in vitro* experiments and, based on these, developed a ccRCC prognostic characterization model, which predicts the prognostic risk of patients with ccRCC, the efficacy of immunotherapy and chemotherapy, and the role of lncRNAs tumor immune infiltration. The risk profile identified in this study not only reveals the role of the combination of lncRNAs that synergistically regulate glycolysis and immunotherapeutic efficacy in ccRCC, but also accurately evaluates patient prognosis in ccRCC and identifies patient populations that would benefit from immunotherapy, providing valuable insights and therapeutic targets for the clinical management of ccRCC.

## Supplementary Material

Supplementary Figures
