# Supplementary material for: Comprehensive multi-omics analysis reveals a combination of lncRNAs that synergistically regulate glycolysis and immunotherapeutic effects in renal clear cell carcinoma
Source: Aging (Albany NY). 2024 Aug 19;16(16):11955–69. doi: 10.18632/aging.206069 (PMC11386928; doi:10.18632/aging.206069)
Supplement: Supplementary Figures [file aging-16-206069-s001.pdf]

SUPPLEMENTARY FIGURES

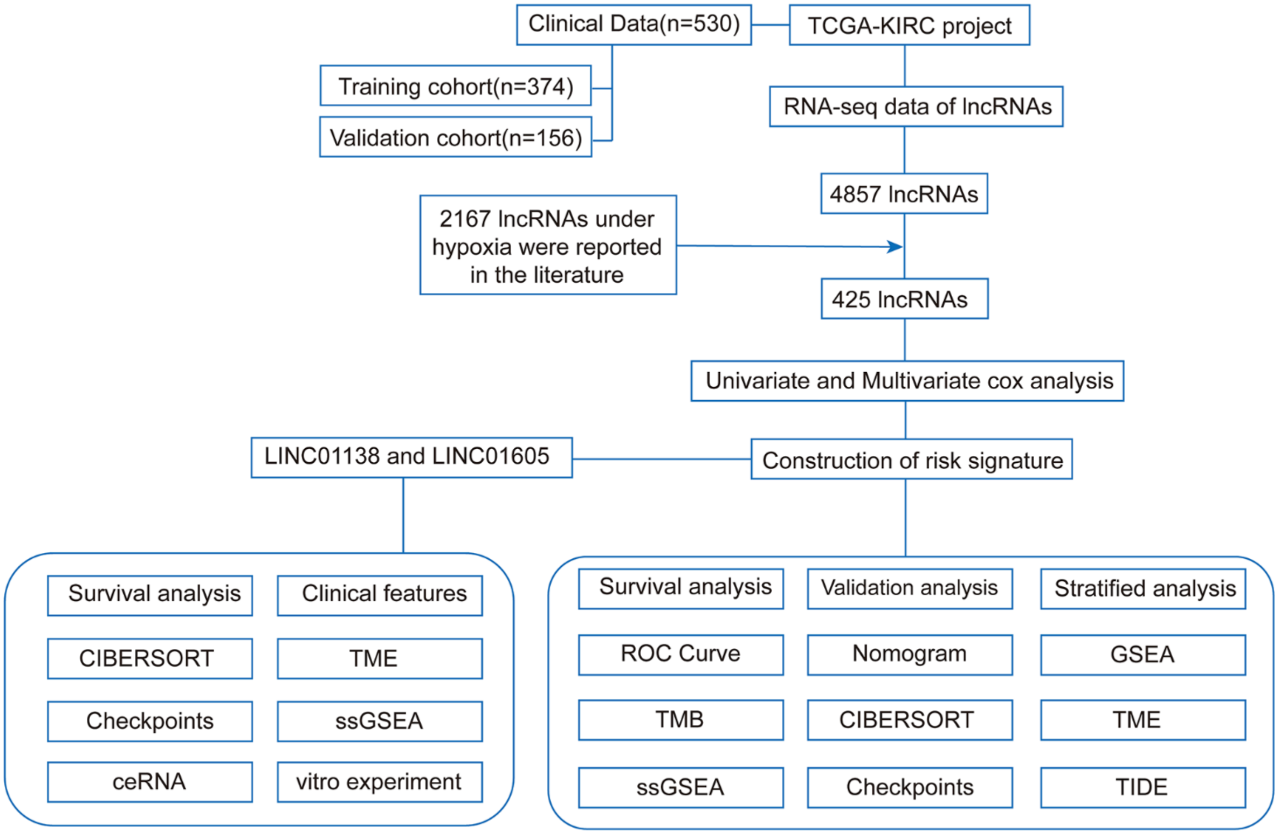

Supplementary Figure 1. The technology roadmap for this study.

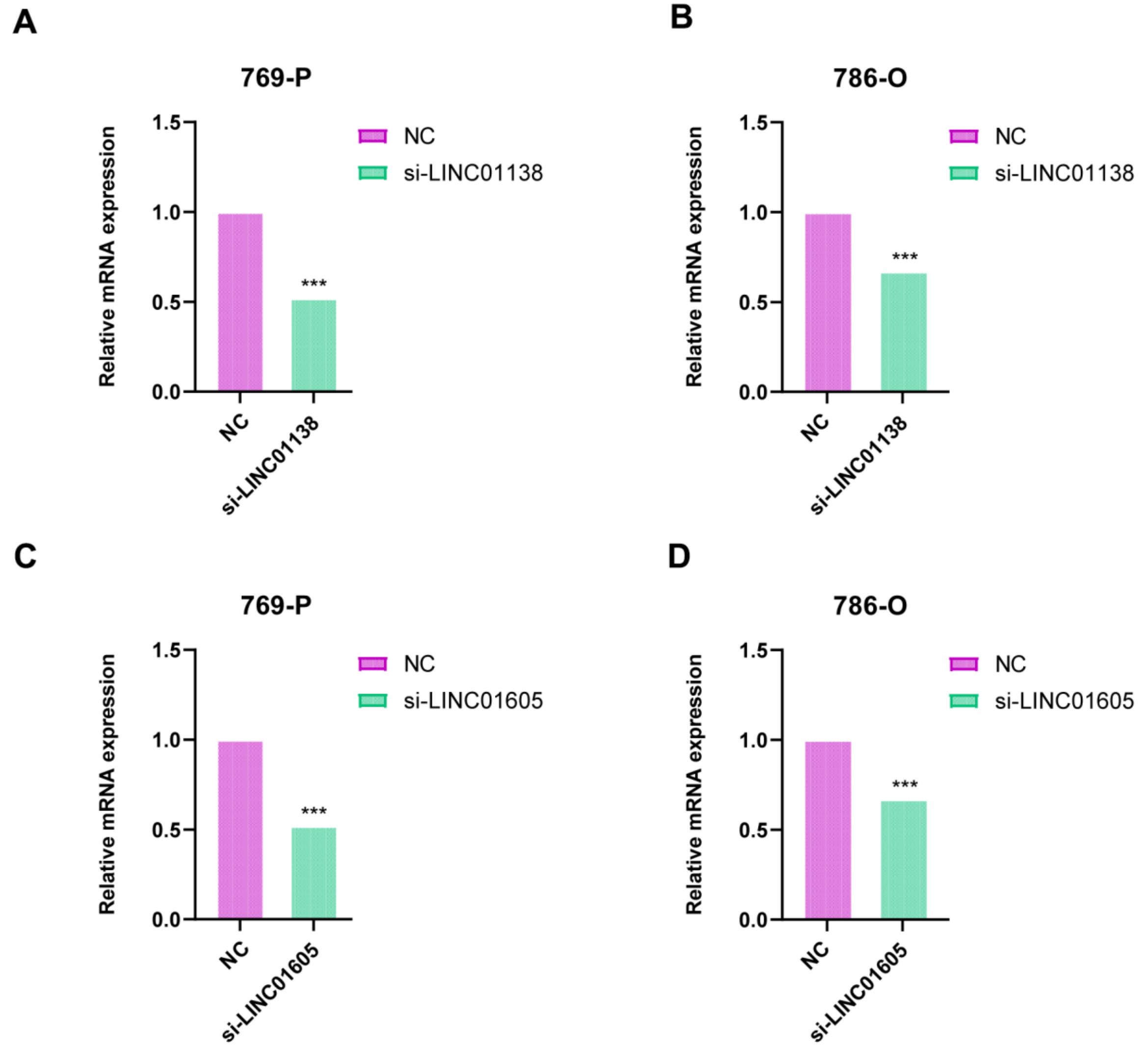

Supplementary Figure 2. (**A**–**D**) QPCR verified the mRNA expression level of LINC01138 and LINC01605 in 769-P and 786-O cells after transfection with siRNA.
